# Cofilin1 oxidation links oxidative distress to mitochondrial demise and neuronal cell death

**DOI:** 10.1038/s41419-021-04242-1

**Published:** 2021-10-16

**Authors:** Lena Hoffmann, Marcel S. Waclawczyk, Stephan Tang, Eva-Maria Hanschmann, Manuela Gellert, Marco B. Rust, Carsten Culmsee

**Affiliations:** 1 Institute for Pharmacology and Clinical Pharmacy, Biochemical-Pharmacological Center Marburg, University of Marburg, Karl-von Frisch Straße 2, 35043 Marburg, Germany; 2 Center for Mind, Brain and Behavior—CMBB, Hans-Meerwein-Straße 6, 35032 Marburg, Germany; 3 Department of Neurology, Heinrich-Heine University Düsseldorf, Moorenstraße 5, 40225 Düsseldorf, Germany; 4 Institute for Medical Biochemistry and Molecular Biology, University Medicine Greifswald, Ferdinand-Sauerbruch-Straße, 17475 Greifswald, Germany; 5 Molecular Neurobiology Group, Institute of Physiological Chemistry, Biochemical-Pharmacological Center Marburg, University of Marburg, Karl-von Frisch Straße 2, 35043 Marburg, Germany; 6 DFG Research Training Group “Membrane Plasticity in Tissue Development and Remodeling”, GRK 2213, University of Marburg, 35032 Marburg, Germany; 7 Third Affiliated Hospital, Zhengzhou University, Zhengzhou, China

**Keywords:** Apoptosis, Cell death in the nervous system, Neurodegeneration

## Abstract

Many cell death pathways, including apoptosis, regulated necrosis, and ferroptosis, are relevant for neuronal cell death and share common mechanisms such as the formation of reactive oxygen species (ROS) and mitochondrial damage. Here, we present the role of the actin-regulating protein cofilin1 in regulating mitochondrial pathways in oxidative neuronal death. Cofilin1 deletion in neuronal HT22 cells exerted increased mitochondrial resilience, assessed by quantification of mitochondrial ROS production, mitochondrial membrane potential, and ATP levels. Further, cofilin1-deficient cells met their energy demand through enhanced glycolysis, whereas control cells were metabolically impaired when challenged by ferroptosis. Further, cofilin1 was confirmed as a key player in glutamate-mediated excitotoxicity and associated mitochondrial damage in primary cortical neurons. Using isolated mitochondria and recombinant cofilin1, we provide a further link to toxicity-related mitochondrial impairment mediated by oxidized cofilin1. Our data revealed that the detrimental impact of cofilin1 on mitochondria depends on the oxidation of cysteine residues at positions 139 and 147. Overall, our findings show that cofilin1 acts as a redox sensor in oxidative cell death pathways of ferroptosis, and also promotes glutamate excitotoxicity. Protective effects by cofilin1 inhibition are particularly attributed to preserved mitochondrial integrity and function. Thus, interfering with the oxidation and pathological activation of cofilin1 may offer an effective therapeutic strategy in neurodegenerative diseases.

## Introduction

Specific redox-active second messengers, such as H_2_O_2_, and specific oxidoreductases of the Thioredoxin (Trx) family of proteins catalyze the regulated oxidation and reduction of Cys residues—so-called thiol switches. Thereby they posttranslationally regulate the function of proteins. In fact, redox regulation is part of signal transduction and is essential for cellular functions [1, 2]. Oxidative distress, i.e., the disruption of redox regulation, can induce neuronal cell death and is widely considered as a pivotal cause of regulated cell death (RCD) in neurodegenerative disorders, such as Alzheimer’s (AD) or Parkinson’s disease (PD) [3–5]. It is widely accepted that major steps of the cell death cascade comprise a detrimental accumulation of intracellular calcium and the formation of reactive oxygen species (ROS) [3, 6] and converge at the level of mitochondria [7, 8]. Mitochondria are dynamic organelles regulating energy metabolism, calcium homeostasis, and the cellular redox balance [7–9]. Thus, mitochondrial demise, including mitochondrial calcium overload, loss of the mitochondrial membrane potential, accumulation of reactive oxygen species, and release of apoptosis-inducing factor (AIF) are considered as the “point of no return” upon cell death induction [8, 10, 11].

In this study, regulated cell death was induced by glutamate or erastin treatment leading to cell death mechanisms called oxytosis or ferroptosis, respectively. Oxytosis is a well-established form of regulated cell death occurring during neuronal development, as well as under pathological conditions in neurodegenerative diseases [12]. In addition, ferroptosis was defined more recently as an iron-dependent form of oxidative cell death, which can be achieved, e.g., by erastin treatment in neuronal HT22 cells [13–15]. Both forms of cell death are mediated through inhibition of the cystine-glutamate (X_C_^-^)-antiporter and reduced glutathione levels resulting in the impaired activity of the glutathione peroxidase-4 (Gpx4), activation of 12/15-lipoxygenase (LOX), and accumulation of ROS [14–17]. Concomitantly, dynamin-related protein 1 (DRP1) and BID are activated and accelerate mitochondrial outer membrane permeabilization (MOMP) [14, 18–20]. Finally, cytochrome c and apoptosis-inducing factor (AIF) are released from mitochondria mediating the degradation of DNA [16, 17, 21].

Cofilin1 is a member of the ADF/cofilin family of actin-depolymerizing proteins and the major representative of this family in neurons [22]. Upon dephosphorylation of a serine residue at position 3 (Ser3) of the protein, it can bind to filamentous actin (F-actin) and initiate its depolymerization [23]. Moreover, it can bind to globular actin monomers (G-actin) and inhibit the nucleotide exchange from ADP-actin to ATP-actin, which is required for F-actin assembly [24]. Thus, cofilin1 can exert indirect effects on molecular mechanisms by operating on actin dynamics and it can also act as a direct participator in the apoptotic cell death cascade by recruitment of cofilin1 from the cytosol to mitochondria [25]. Importantly, cofilin1’s effects on mitochondria can be versatile, as it was shown to be involved in transducing apoptotic signaling to mitochondria upon oxidation [25, 26]. Oxidation of cofilin1 is an important posttranslational modification for the regulation of cytoskeletal dynamics (reviewed in ref. [27]). Moreover, acting on mitochondrial dynamics via DRP1 activation was demonstrated in a cofilin1 loss-of-function approach in mouse fibroblasts [28].

The results obtained in this study demonstrate a role for cofilin1 as a redox sensor and regulator of oxytosis or ferroptosis upstream of mitochondria in neuronal HT22 cells, and after glutamate-induced excitotoxicity in primary neurons.

## Materials and methods

### Cell culture

HT22 cells originate from immortalized primary mouse hippocampal neurons. HT22 cells were incubated at 37 °C and 5% CO_2_ in Dulbecco’s modified Eagle’s high glucose medium (DMEM; Sigma-Aldrich, Munich, Germany) supplemented with 10% fetal bovine serum, 20 mM HEPES, 100 units/mL penicillin, 100 µg/mL streptomycin, and 2 mM glutamine.

For efficient cofilin 1 knockdown, HT22 cells were transfected with 15 nM cofilin1 siRNA for 48 h using Lipofectamine RNAiMAX (Thermo Fisher Scientific, Darmstadt, Germany) according to the manufacturer. Following siRNA sequences were obtained from Dharmacon: scrambled siRNA (scrsiRNA, 5′-UAAUGUAUUGGAACGCAUA-3′), *Cofilin1* siRNA1 (CflsiRNA1, 5’-AGACAAGGACUGCCGCUAU-3’) and *Cofilin1* siRNA2 (CflsiRNA2, 5’-GGAAUCAAGCAUGAAUUAC-3’). The sensitivity of HT22 cells to glutamate highly depends on cell density and on cysteine-glutamate antiporter (xCT) expression levels. In this biological system, glutamate concentrations are usually adjusted between 5 and 10 mM to achieve 70–80% cell damage. Erastin toxicity also depends on cell density and xCT expression, and also needs adjustment within a concentration range between 0.7 and 3 µM.

Primary cortical neurons were prepared from embryonic mouse brains (E18) as described previously [21]. Genetically modified mice expressing cofilin1 allele with exon 2 flanked by loxP sites were used as controls (Ctrl) [29]. Cofilin1 knockout was achieved by expression of the Cre enzyme capable of recognizing loxP sites and thus specifically deleting exon 2 of the cofilin1 gene region, resulting in a nonfunctional gene product. Since a systemic knockout of cofilin1 is embryonically lethal [30], Cre expression is under the control of a CaMKIIα promotor to specifically delete cofilin1 in excitatory neurons for the forebrain-including cerebral cortex neurons [31]. Neuronal cultures were grown in neurobasal medium (Thermo Fisher Scientific, Darmstadt, Germany) supplemented with 1.2 mM glutamine, 2% B27 Plus supplement (Thermo Fisher Scientific, Darmstadt, Germany), 100 U/mL penicillin, and 100 µg/mL streptomycin. Glutamate treatment (25 μM) was conducted at day 30 in culture (DIV30) for 24 h. NMDA-antagonist MK801 (Merck KGaA, Germany) was added as a control at a concentration of 10 μM simultaneously to glutamate addition. Rho-activator II CN03 (Cytoskeleton, Denver, USA) was applied at a concentration of 1 µg/mL 3 h prior to glutamate treatment.

### RT-(q)PCR

Total RNA was isolated by using InviTrap Spin Universal RNA Mini Kit (Stratec Biomedical, Birkenfeld, Germany) 48 h after siRNA transfection. SuperScript III One-Step RT-PCR System (Thermo Fisher Scientific, Darmstadt, Germany) was used to perform reverse transcription PCR (RT-PCR) and specific oligonucleotides (*Gapdh* (399 bp) forward 5′-CGTCTTCACCACCATGGAGAAGGC-3′ and reverse 5′-AAGGCCATGCCAGTGAGCTTCCC-3′ and *Cofilin1* (146 bp) forward 5’-GCCAACTTCTAACCACAATAG-3’ and reverse 5’-CCTTACTGGTCCTGCTTCC-3’). The amplification products were visualized by agarose gel electrophoresis after staining with ethidium bromide by illumination with UV light.

Quantitative PCR was conducted in the StepOnePlus Real-Time PCR System (Fisher Scientific GmbH, Schwerte, Germany). mRNA was extracted using the InviTrap Spin Universal RNA Mini Kit (Invitek Molecular GmbH, Berlin, Germany) according to the manufacturer’s protocol and treated with Turbo DNA-free Kit. cDNA synthesis was conducted with 200 ng of DNAse-treated RNA via iScript™ cDNA Synthesis Kit (Bio-Rad Laboratories GmbH, Munich, Germany) and then diluted 1:5 with aqua bidest and added to a mastermix containing iTaq Universal SYBR Green Supermix (Bio-Rad Laboratories GmbH, Munich, Germany). The respective primer pair for the genes of interest in a MicroAmp™ Fast Optical 96-Well Reaction Plate (Thermo Fisher Scientific, Waltham, MA, USA). Relative gene expression levels were analyzed by double-delta C_T_ analysis and shown as 2^-ΔΔCτ^. The following oligonucleotides were used for RT-qPCR measurements: Cofilin1 forward (AGGACCTGGTGTTCATCTTCTG), Cofilin1 reverse (TGCTTGATTCCTGTCAGCTTCT), GAPDH forward (CCCTTCATTGACCTCAACTA), GAPDH reverse (CCAAAGTTG TCATGGATGAC), U6 forward (CTCGCTTCGGCAGCACA), U6 reverse (AACGCTT CACGAATTTGCGT).

### Protein analysis

Protein extraction and Western blot analysis were performed as previously described [11]. Briefly, cells were ruptured in a lysis buffer containing 0.25 M d-mannitol, 0.05 M Tris base, 1 mM EDTA, 1 mM EGTA, 1 mM DTT, and 1% Triton X-100 supplemented with protease and phosphatase inhibitor cocktail tablets (Roche Diagnostics, Mannheim, Germany). The total protein amount was determined using the Pierce BCA Protein Assay Kit (Thermo Fisher Scientific, Darmstadt, Germany). Proteins were separated by gel electrophoresis and then transferred onto a PVDF membrane (Roche Diagnostics, Mannheim, Germany). Cofilin1 and phospho-Cofilin1 (Ser3) (1:1000; Cell Signaling Technology, Danvers, USA), as well as α-tubulin as a loading control, were analyzed using indicated primary antibodies (α-tubulin antibody 1:10,000; Sigma-Aldrich, Munich, Germany). After incubation with the appropriate secondary HRP-labeled antibody (Vector Laboratories, Burlingame, CA, USA), Western blot signals were detected by chemiluminescence with Chemidoc system (Bio-Rad, Munich, Germany).

### Cell viability

Cell viability was assessed by a colorimetric assay based on the yellow-colored MTT reagent (3-(4,5-dimethyl-2-thiazolyl)-2,5-diphenyl-2H-tetrazolium bromide, 0.5 mg/mL for HT22 cells and 1 mg/mL for primary cortical neurons; Sigma-Aldrich, Munich, Germany) which is reduced to a purple-colored formazan quantified by absorbance measurement at 570 nm with a reference filter at 630 nm by FluoStar OPTIMA reader (BMG Labtech, Ortenberg, Germany). Cell death was monitored in real-time using the xCELLigence Real-Time Cell Analysis (RTCA; Roche Diagnostics, Mannheim, Germany) system as previously described [32]. Changes in the impedance are depicted as normalized cell index.

### Flow cytometry

Different cellular and mitochondrial parameters of the glutamate- or erastin-induced cell death pathways were analyzed using the Guava easyCyte 6–2 L flow cytometer (Merck Millipore, Darmstadt, Germany) upon harvesting adherent HT22 cells and following the addition of different fluorescent dyes. Apoptotic and late necrotic cells were identified using the Annexin V-FITC Detection Kit (Promokine, Heidelberg, Germany). Annexin V and propidium iodide (PI) staining were performed for 5 min in the dark at room temperature after harvesting the cells with trypsin. By staining the cells with BODIPY 581/591 C11 (4,4-difluoro-5-(4-phenyl-1,3-butadienyl)-4-bora-3a,4a-diaza-s-indacene-3-undecanoic acid; Thermo Fisher Scientific, Darmstadt, Germany) oxidized lipids and membranes were detected. Following 8 h of glutamate treatment, the cells were stained with 2 µM BODIPY dye for 1 h at 37 °C. The cell-permeable dye 2’,7’-dichlorodihydrofluorescein diacetate (H_2_DCF-DA) was used to evaluate the accumulation of cellular reactive oxygen species upon 30 min incubation of 20 µM DCF dye in DMEM without serum. For evaluation of mitochondrial reactive oxygen species accumulation, MitoSOX Red indicator (Thermo Fisher Scientific, Darmstadt, Germany) was applied at a concentration of 1.25 µM for 30 min at 37 °C. For the MitoSOX measurement with isolated mitochondria, 10 µM of the complex III-inhibitor antimycin A (AA) was used as a positive control and incubated simultaneously with MitoSOX Red indicator at a concentration of 1.25 µM and afterward measured with Guava easyCyte 6–2 L flow cytometer (Merck Millipore, Darmstadt, Germany). Mitochondrial membrane potential was measured upon staining the cells with MitoPT TMRE Kit (ImmunoChemistry Technologies, Hamburg, Germany). Therefore, cells were incubated with 0.2 µM TMRE (tetramethylrhodamine ethyl ester) for 30 min at 37 °C. For investigation of the mitochondrial membrane potential of isolated mitochondria from adult mouse brain tissue, 40 µg mitochondria were diluted in 150 µL 1× mitochondrial assay solution (MAS: 70 mM sucrose, 220 mM mannitol, 10 mM KH_2_Cl_2_, 5 mM MgCl_2_, 2 mM HEPES, 1 mM EGTA, 0.20% BSA, pH 7.2) supplemented with 2 µM rotenone and 10 mM succinate. In total, 50 µM of the uncoupler CCCP was used as a positive control. Mitochondria were incubated with 0.2 µM TMRE for 15 min and then measured with Guava easyCyte 6–2 L flow cytometer (Merck Millipore, Darmstadt, Germany). Staining the cells with the mitochondrial selective dye Rhod-2 AM (rhodamine-2 acetoxymethyl ester; Thermo Fisher Scientific, Darmstadt, Germany), allows for specific evaluation of mitochondrial calcium accumulation. Therefore, Rhod-2 AM was reduced to Dihydrorhod-2 AM and incubated at a concentration of 2 µM in DMEM without serum for 1 h.

### Measurement of ATP, mitochondrial oxygen consumption rate (OCR), and extracellular acidification rate (ECAR)

Cellular ATP levels were measured using the ViaLight Plus Kit (Lonza, Verviers, Belgium) according to the manufacturer’s protocol. Briefly, cells were lysed, transferred to a white-walled 96-well plate, and the ATP monitoring reagent was added to the cell lysate. Afterward, the luminescence was detected with a FLUOstar OPTIMA reader (BMG Labtech, Ortenberg, Germany). Determination of the mitochondrial oxygen consumption rate as an indicator of mitochondrial respiration was performed using the Seahorse XFe96 Analyzer (Agilent Technologies, Waldbronn, Germany). Cells were plated in XFe96-well microplates (6000 cells/well, Seahorse Bioscience), and 1 h prior to the measurement, the growth medium was replaced by the seahorse assay medium (4.5 g/l glucose, 2 mM glutamine, 1 mM pyruvate, pH 7.35). After recording three baseline measurements, four compounds were added by injection.

The compounds and final concentrations used are as follows: oligomycin 3 µM, FCCP 0.5 µM, rotenone 0.1 µM, and antimycin A 1 µM. For measuring mitochondrial respiration, 5–12 µg of isolated mitochondrial protein was measured in 1×MAS containing the complex II substrate succinate (10 mM) and the complex I inhibitor rotenone (2 µM) to focus on mainly complex II- and complex III-driven respiration. For slight attachment of the plated mitochondria at the bottom of the cell plate, a centrifugation step of the whole plate at 2000×*g* for 20 min at 4 °C was indispensable (Heraeus Megafuge 40 R; Thermo Fisher Scientific, Darmstadt, Germany). Indicated compounds for the injections were used in the following final concentrations: 4 mM ADP, 2.5 µg/mL oligomycin, 4 µM FCCP, and 4 µM antimycin A (AA). Three basal and three measurements after each injection were recorded with the Seahorse XFe96 Analyzer (Agilent Technologies, Waldbronn, Germany).

### Mitochondrial isolation

Mitochondrial isolation of freshly dissected cortical or hippocampal brain tissue (~50 mg) was performed as previously described [21]. The tissue was embedded in 2 mL mitochondrial isolation buffer (composed of 300 mM sucrose, 5 mM TES, 200 µM EGTA, pH 7.2) and roughly homogenized with a 20-G Neoject needle (Dispomed, Gelnhausen, Germany) and then sieved through a 100-µm nylon cell strainer (Corning Incorporated, Corning, NY, USA). To homogenize the tissue efficiently and extract mitochondria from the cell structure thoroughly, a cell homogenizer (Isobiotec, Heidelberg, Germany) with 1 mL gas-tight syringes (Supelco, Munich, Germany) was used to ensure a constant rate of 700 µL/min. The cell homogenizer contained a spherical tungsten carbide ball with a clearance of 10 µm to decompose the tissue but simultaneously maintain the integrity of mitochondria. The cell homogenate was transferred into 1.5 mL tubes and centrifuged at 800 x *g* for 10 min at 4 °C to remove cell debris. Afterward, the supernatant was transferred into a fresh tube and centrifuged at 10,000 x *g* again for 10 min at 4 °C (Heraeus™ Fresco™ 17 Mikrozentrifuge; Thermo Fisher Scientific, Darmstadt, Germany). The resulting pellet consists of the crude mitochondrial fraction, which was finally resuspended in MSHE-BSA (composed of 70 mM sucrose, 210 mM mannitol, 5 mM HEPES, 1 mM EGTA, 0.5% (w/v) BSA, pH 7.2) buffer. The seahorse measurement was performed in mitochondrial assay solution, composed of 70 mM sucrose, 220 mM mannitol, 10 mM KH_2_PO_4_, 5 mM MgCl_2_, 2 mM HEPES, 1 mM EGTA, 0.2 % (w/v) BSA, pH 7.2). All steps were performed on ice or at 4 °C. Pierce™ BCA Kit was used to determine the protein amount of the mitochondrial fraction.

### Protein expression and purification

Human cofilin1 was amplified by PCR using specific oligonucleotides (forward: 5’-CATATGGCCTCCGGTGTG-3’, reverse: 5’-GGATCCTCACAAAGGCTTGCCCTC-3’) and cloned into the pET-15b vector (Novagen, Millipore, UK). By using site-directed mutagenesis, the cysteine residues of cofilin1 were mutated into serine residues using complementary oligonucleotides harboring the nucleotide exchanges (Cys39Ser: forward: 5’-GTGCTCTTCTCCCTGAGTG-3’, reverse: 5’-CACTCAGGGAGAAGAGCAC-3’; Cys80Ser: forward: 5’-CATAAGGACTCCCGCTATGC-3’, reverse: 5’-GCATAGCGGGAGTCCTTATC-3’; Cys139Ser: forward: 5’-CAAGCAAACTCCTACGAGGAG-3’, reverse: 5’-CTCCTCGTAGGAGTTTGCTTG-3’; Cys147Ser: forward: 5’-GACCGCTCCACCCTGG-3’, reverse: 5’-CCAGGGTGGAGCGGTC-3’) and the KOD Hot Start Mastermix (Merck, Darmstadt, Germany). The plasmids were confirmed by sequencing (Seqlab, Göttingen, Germany).

The human cofilin1 WT, the mutant lacking two Cys residues (Cys139/147Ser, 2Cys → Ser), and the mutant lacking all four Cys residues (4Cys → Ser) were expressed as His-Tag fusion proteins in *E. coli* as described before [33]. The proteins were purified by immobilized metal affinity chromatography using the His Trap Kit from GE Healthcare Life Science, USA. Expression and purification efficiency were analyzed by SDS-PAGE using precast gels from Bio-Rad, USA, and Coomassie staining. Proteins were re-buffered into PBS using Zeba Spin columns and the thermal stability of the proteins was analyzed by recording the emission at 600 nm over time with increasing temperature from 20 to 70 °C (2 °C per 3 min) using the Shimadzu UV1800.

Recombinant cofilin1 was either used in a native way, oxidized by 100 µM H_2_O_2_ incubation for 30 min, or reduced with 10 mM freshly dissolved dithiothreitol (DTT) for 30 min. The remaining elution buffer from the protein purification process was substituted by PBS using sephadex-based PD MidiTrap G-25 columns (GE Healthcare, Chicago, USA). Afterward, protein amount was determined by a NanoPhotometer™ (Implen, Munich, Germany). The experiments were performed using 0.13–0.25 µg recombinant protein per µg mitochondrial protein and incubated for 30–60 min at room temperature and another 10 min at 37 °C and afterward measured as indicated at the respective method.

## Results

### Cofilin1 downregulation attenuates glutamate- and erastin-induced cell death

**Fig. 1:**
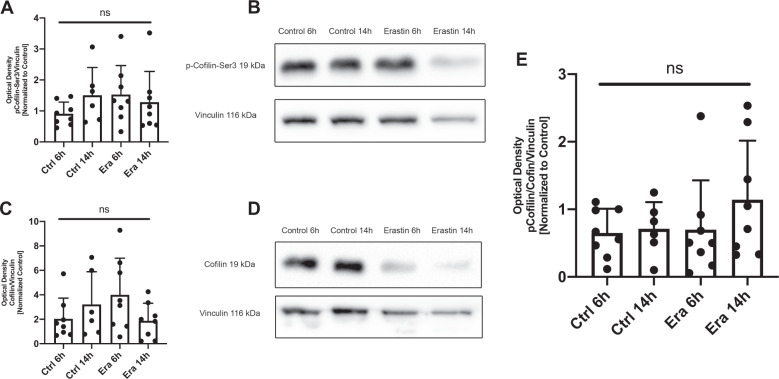
Cofilin phosphorylation status is not altered by glutamate or erastin challenge. **A**–**D** HT22 cells were treated with 1 µM erastin and the respective amount of DMSO for the indicated time points for Western blot analysis. Six to eight independent blots of phosphorylated cofilin-Ser3 (1:1000) (**A**) as well as total cofilin1 (1:1000) (**C**) were normalized to respective vinculin loading controls (1:20,000) and quantified (mean + standard deviation, SD). Respective representative blots are shown for phosphorylated Cofilin-Ser3 (**B**) and total cofilin1 (**D**). **E** The quantification of the activation of cofilin1 in HT22 cells challenged with 1 µM erastin to the indicated time points are evaluated (mean + SD). Ns non-significant to control (one-way ANOVA, Bonferroni’s post hoc test).

The phosphorylation and oxidation state of cofilin1 determines not only its binding capacity to F-actin [34–36], it is also essential for translocation to mitochondria [26]. In particular, dephosphorylated cofilin1 attains activity for translocation from the cytosol to mitochondria [37]. In western blot analyses, we detected reduced cofilin expression and cofilin1 phosphorylation in some of the samples at 14 h after the induction of ferroptosis in the HT22 cells. However, these effects showed a high variability due to cell death after induction of ferroptosis, and overall no significant differences between the groups were detected, when correcting for the vinculin signal (Fig. 1A–E). Similarly, mRNA expression levels of cofilin1 after exposure to glutamate and erastin were not altered at the different time points, suggesting that cofilin1 was not significantly regulated after oxytosis or ferroptosis, respectively (Supplementary Fig. 1S).

**Fig. 2:**
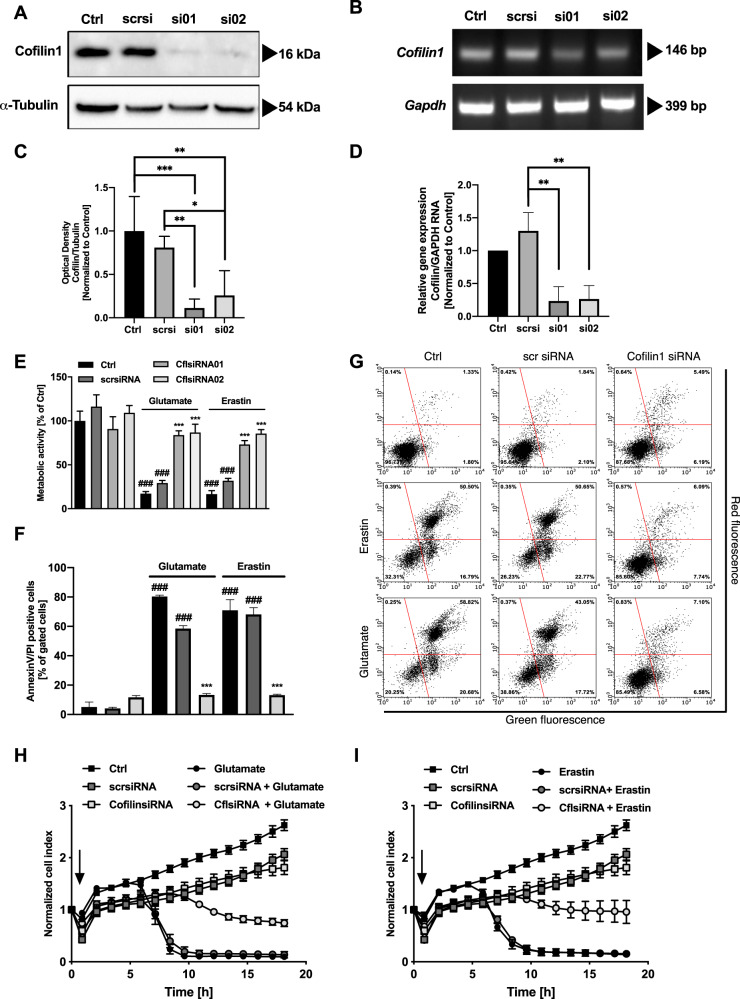
Cofilin1 depletion reveals sustained protection after glutamate or erastin exposure which occurs independently of lipid peroxidation and ROS accumulation. HT22 cells were transfected with two specific siRNAs against cofilin1 showing comparable effects. Unspecific scrambled siRNA (scrsi) was used as control. Transfection efficiency was analyzed after 48 h on (**A**, **C**) protein and (**B**, **D**) mRNA level. Protein levels were determined by western blot using specific antibodies against cofilin1 and α-tubulin as a loading control, shown as mean + SD (*n* = 4 replicates). ***P < 0.001; **P < 0.01; *P < 0.05 (one-way ANOVA, Bonferroni’s post hoc test). Transfection efficiency was also analyzed on *mRNA* level by (**B**) RT-PCR and (**D**) RT-qPCR. *Gapdh* was used as an internal control. Bands are cropped at the indicated size for better comprehension with ImageLab software (Bio-Rad, California, USA). **D** mRNA expression levels of RT-qPCR are shown as mean + SD (*n* = 3 replicates); **P < 0.01 (one-way ANOVA, Bonferroni’s post hoc test). **E** Cells were treated with 0.2 µM erastin or 2 mM glutamate for 16 h and were analyzed for proliferation/viability using MTT reagent. Both siRNA sequences conveyed comparable effects, therefore subsequent experiments were performed with siRNA1. Values are shown as mean + SD (*n* = 8 replicates). **F**, **G** Annexin V and PI staining was conducted after 30 h of siRNA incubation and following 16 h of erastin (0.5 µM) or glutamate (2 mM) treatment (mean + SD; 5000 cells per replicate of *n* = 3 replicates). **H**, **I** xCELLigence measurement was performed after siRNA incubation for 30 h. The arrow indicates the time of erastin (0.75 µM) or glutamate (8 mM) application. Data are given as mean ± SD (*n* = 8 replicates). ^###^*P* < 0.001 compared to untreated ctrl; ***P < 0.001 compared to erastin- or glutamate-treated ctrl (ANOVA, Scheffé’s test).

Next, we specified the importance of cofilin1 in a loss-of-function approach using two different siRNA sequences (si01, si02) for 48 h. The knockdown efficacy was detected by Western blot and RT-(q)PCR (Fig. 2A–D). At the protein level, Western blot analysis revealed an efficient knockdown of cofilin1 (Fig. 2A, C). Knockdown of cofilin1 prevented cell damage after 16 h of erastin or glutamate exposure (Fig. 2E). Further, deletion of cofilin1 reduced the number of Annexin V and propidium iodide (PI)-positive cells upon erastin or glutamate exposure (Fig. 2F, G). The xCELLigence real-time cell analysis (RTCA) confirmed the protective effects of cofilin1 silencing against oxidative death in living cells. Within 5–10 h of treatment, untransfected controls and cells transfected with the scrambled siRNA detached, resulting in declined cell index. Cofilin1-depleted cells were rescued (Fig. 2H, I). These observations demonstrate a relevant involvement of cofilin1 in oxidative cell death of oxytosis and ferroptosis.

### Cofilin1 acts downstream of lipid peroxidation and cellular reactive oxygen species formation, but upstream of mitochondrial demise

**Fig. 3:**
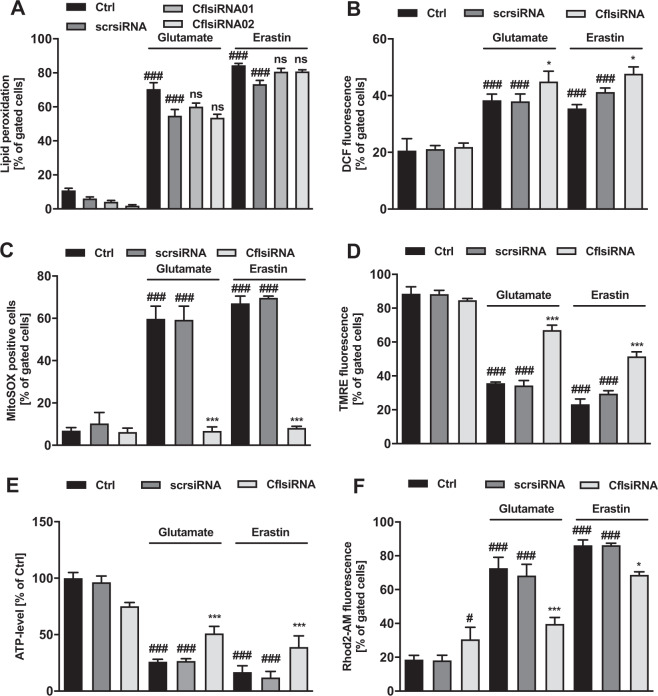
Cofilin1 silencing is effectively averting mitochondrial impairment in terms of glutamate or erastin toxicity. Prior to the described measurement, cofilin1 siRNA was incubated for 30 h. **A** Lipid peroxidation was determined 9 h after challenging the cells with 0.5 µM erastin or 5 mM glutamate with BODIPY fluorescent dye and subsequent FACS measurement. Data are given as mean + SD; 5000 cells per replicate of *n* = 3 replicates. **B** The amount of ROS was measured after 0.8 µM erastin or 7 mM glutamate treatment for 10 h following H_2_DCF-DA staining and FACS analysis. Data are given as mean + SD; 5000 cells per replicate of *n* = 3 replicates. **C** Mitochondrial superoxide accumulation was measured by MitoSOX staining and FACS analysis after 16 h treatment with 0.5 µM erastin or 4 mM glutamate. Data are presented as mean + SD; 5000 cells per replicate of *n* = 3 replicates. **D** The mitochondrial membrane potential was evaluated by an appropriate cell-permeant, positively charged TMRE dye and following FACS analysis after treatment for 16 h with 1 µM erastin or 10 mM glutamate. Data are given as mean + SD; 5000 cells per replicate of *n* = 3 replicates. **E** Cells were challenged for 8 h with 0.7 µM erastin or 7 mM glutamate. ATP content was measured by luminescence-based measurement. Values are shown as mean + SD (*n* = 8 replicates. **F** Rhod-2 acetoxymethyl ester (Rhod-2 AM) was used to specifically measure mitochondrial calcium level after 16-h treatment with 0.8 µM erastin or 8 mM glutamate. Values are displayed as mean + SD; 5000 cells per replicate of *n* = 3 replicates. Ctrl (control); scrsi (scrambled siRNA); Cfl1si (cofilin1 siRNA), ^#^*P* < 0.05 and ^###^*P* < 0.001 compared to untreated ctrl, *P < 0.05 compared to erastin- or glutamate-treated ctrl, ***P < 0.001 compared to erastin- or glutamate-treated ctrl, ns not significant (ANOVA, Scheffé’s test).

To further validate the specific activity of cofilin1, lipid peroxidation was assessed using the fluorescent dye BODIPY C11 and flow cytometry [38]; ROS were assessed using H_2_-DCF. Of note, both erastin and glutamate treatment-induced pronounced accumulation of lipid peroxides, which was not affected by siRNA-mediated cofilin1 knockdown (Fig. 3A), indicating that protective effects exerted by cofilin1 knockdown occur downstream of lipid peroxidation. Interestingly, cofilin1 depletion could not diminish the fluorescent DCF signal (Fig. 3B), thus, in the applied model systems of oxidative cell death, protective effects exerted by cofilin1 knockdown occur downstream of cellular ROS generation. Next, we used the MitoSOX marker that specifically detects mitochondrial superoxide. Both glutamate and erastin challenge triggered massive mitochondrial superoxide accumulation, however, cofilin1 depletion completely blocked such mitochondrial superoxide formation (Fig. 3C). Further, loss of the mitochondrial membrane potential assessed by TMRE staining was considerably impaired after exposure to oxidative challenges, and this was attenuated by cofilin1 knockdown (Fig. 3D). Cofilin1 depletion alone neither impaired mitochondrial ROS generation nor the mitochondrial membrane potential. Further, glutamate and erastin treatments resulted in an overall decline of ATP levels, while cofilin1 depletion partly rescued ATP production (Fig. 3E). Massive mitochondrial calcium accumulation is considered as a detrimental prerequisite for mitochondrial impairment, eventually provoked by increased calcium-induced mitochondrial respiration, nitric oxide production, and finally, loss of mitochondrial membrane integrity [39]. To address mitochondrial calcium alterations, Rhod-2 AM staining and flow cytometry measurements were performed. The results revealed that cofilin1 depletion could significantly reduce the massive mitochondrial calcium accumulation following glutamate or erastin exposure (Fig. 3F).

**Fig. 4:**
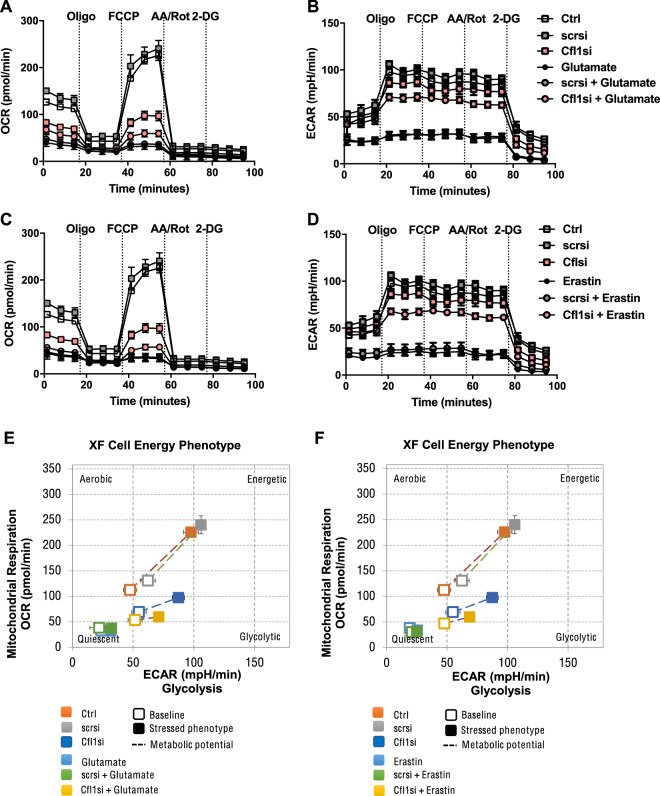
Cofilin1-deficient HT22 cells turn their energy production towards glycolysis after glutamate or erastin exposure. Cofilin1 siRNA was transfected for 48 h. Afterward, cells were damaged for 9 h with 0.5 µM erastin or 7 mM glutamate. **A**, **C** Afterward, the oxygen consumption rate (OCR) and **B**, **D** the extracellular acidification rate (ECAR) were determined by a Seahorse XFe96 Analyzer. Data of 3–6 replicates per condition are shown as mean ± SD. Oligo (oligomycin); FCCP (carbonyl cyanide 4-(trifluoromethoxy)phenylhydrazone); AA (antimycin A) Rot (rotenone); 2-DG (2-deoxy-d-glucose). Ctrl (untransfected control); scrsi (scrambled siRNA); Cfl1si (cofilin1 siRNA). **E**, **F** The cell energy phenotype correlates the OCR and the ECAR of the cells at basal conditions (open dot) measured before the first compound was injected by the system and after FCCP injection, representing a stressed phenotype (filled dot). The displayed metabolic potential (dashed line) represents the capacity to meet the required energy demand under conditions of stress. Ctrl control, scrsi scrambled siRNA, Cfl1si cofilin1 siRNA.

With regard to energy metabolism, the Seahorse XFe96 Analyzer was used to determine both mitochondrial respiration by measuring the oxygen consumption rate (OCR) and glycolysis by measuring the extracellular acidification rate (ECAR). This measurement revealed complete inhibition of mitochondrial respiration and a diminished glycolysis rate after 9 h of glutamate or erastin treatment in neuronal HT22 cells (Fig. 4A–D). Cofilin1 downregulation partly preserved (Glutamate: *scrsi* 36.2 ± 4.7 pmol/min → *Cfl1si* 53.6 ± 8.1 pmol/min; Erastin: *scrsi* 35.7 ± 7.7pmol/min → *Cfl1si* 50.0 ± 5.5 pmol/min) mitochondrial respiration in both paradigms of cellular death (Fig. 4A, C). At earlier time points, basal as well as maximal respiration was reduced in cofilin1-knockdown cells challenged with erastin for 6 h in comparison to non-transfected cells, demonstrating that cofilin1 knockdown exerted effects on energy metabolism at mitochondria independent of the ferroptosis challenge (Supplementary Fig. 2S). In addition, the ability to generate energy by glycolysis was considerably maintained in cofilin1-knockdown cells in oxytosis and ferroptosis (Glutamate: *scrsi* 30.7 ± 5.1 mpH/min → *Cfl1si* 69.9 ± 2.8 mpH/min; Erastin: *scrsi* 28.2 ± 6.3 mpH/min → *Cfl1si 68.6* ± 2.8 mpH/min) (Fig. 4B, D), suggesting a metabolic switch toward glycolytic-based energy production in cofilin1-deficient neurons exposed to oxidative stress. In this regard, the correlation between OCR and ECAR illustrates the metabolic potential of the cells, measured under baseline and stressed conditions by FCCP injection (Fig. 4E, F). Especially after glutamate or erastin treatment, metabolic bioenergetics underwent a mostly quiescent state in control conditions, whereas cells deficient in cofilin1 exhibited a considerably higher metabolic potential, indicating a functional energy production during oxidative stress (Fig. 4E, F). Of note, under control conditions, cofilin1 knockdown itself significantly shifted the metabolic state (Fig. 4E, F, blue box).

### Primary cortical neurons deficient for cofilin1 showed enhanced resilience to glutamate excitotoxicity

**Fig. 5:**
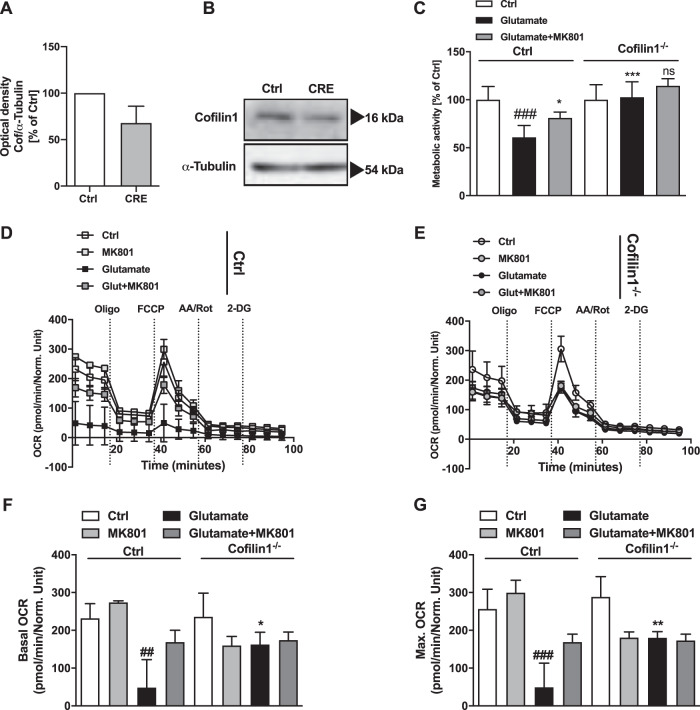
Cofilin1 knockout in primary cortical neurons reveals protection against glutamate-induced excitotoxicity. **A**, **B** Western blot analysis was conducted after 30 *days* in vitro (DIV30) of control (Ctrl) neurons and cofilin1^flx/flx, CaMKIIα-Cre^ neurons (CRE). Protein levels were determined by western blot using specific antibodies against cofilin1 and α-tubulin as a loading control, shown as mean + SEM (*n* = 3 replicates). Bands are cropped at the indicated size for better comprehension with ImageLab software (Bio-Rad, California, USA). **C** Metabolic activity of DIV30 Ctrl and cofilin1^−/−^ neurons was determined by MTT assay after glutamate exposure for 24 h. MK801 co-treatment served as a protected control by NMDA-receptor inhibition. Mean values + SD of *n* = 5 replicates are shown. **D** OCR of Ctrl and **E** cofilin1^−/−^ neurons was measured at 30 days in vitro after 25 µM glutamate challenge for 24 h. 20 µM MK801 was applied simultaneously and served as a control for protective NMDA-R inhibition. **F** Quantification of the basal OCR of Ctrl and cofilin1^−/−^ neurons, measured before the first compound was injected and **G** maximal OCR after FCCP injection of *n* = 3–5 replicates. Mean values ± SD are given. ns (not significant) compared to glutamate-treated cofilin1^−/−^ cells; ^##^*P* < 0.01 and ^###^*P* < 0.0001 compared to untreated ctrl, *P < 0.05; **P < 0.01 and ***P < 0.001 compared to glutamate-treated ctrl (ANOVA, Scheffé’s test).

To confirm the relevance of cofilin1 in primary neurons, we isolated cortical neurons from embryos of cofilin1^flx/flx^ mice 8 (Ctrl) and cofilin1^flx/flx, CaMKIIα-Cre^ regarded as cofilin1 knockout neurons. Western blot analysis confirmed cofilin1 downregulation in Cre-expressing neurons after 30 days in vitro (DIV30) (Fig. 5A,B). MTT assay was used to examine the remaining metabolic activity of cofilin1^−/−^ knockout cells after glutamate exposure in comparison to Ctrl. In Ctrl cells, glutamate treatment led to a significant reduction of metabolic activity, which was prevented by the NMDA-receptor antagonist MK801 (Fig. 5C). In cofilin1-deficient neurons, the detrimental impact of glutamate was entirely abrogated, similar to the effects of MK801 (Fig. 5C).

To gain further insight into the metabolic activity of primary neurons, we performed seahorse measurements. These assays revealed that the oxygen consumption rate, as an indicator of mitochondrial function, was decreased after glutamate exposure of Ctrl neurons under basal conditions and upon evoking maximal respiration by FCCP (Fig. 5D,E). MK801 was able to prevent the detrimental impact of glutamate on basal and maximal OCR (Fig. 5D–G). Similarly, mitochondria of cofilin1 knockout neurons were also significantly protected against glutamate-induced loss of basal and maximal respiration (Fig. 5D–G), indicating that cofilin1 mediated mitochondrial damage in models of glutamate excitotoxicity in the cortical neurons.

**Fig. 6:**
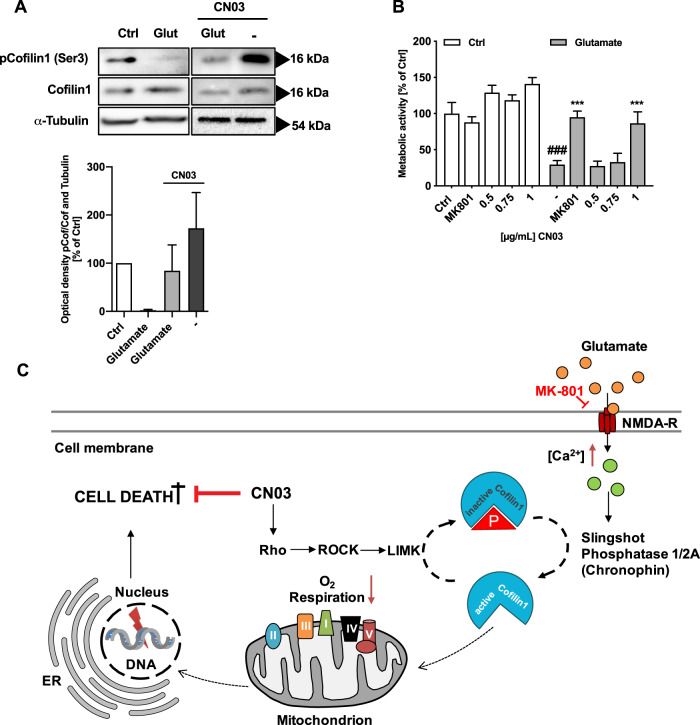
Enhancing cofilin1 phosphorylation reveals neuronal survival after glutamate exposure. **A** Western blot analysis of phosphorylated Ser3-cofilin1 was performed after 3 h pretreatment with 1 µg/mL CN03 and an additional 24 h treatment with 25 µM glutamate. Quantification of the resulting signal was realized by densitometric analysis from *n* = 4 blots. The intensities of pCofilin1 (Ser3) were compared to the cofilin1 signal and to α-tubulin as a loading control and presented as mean + SD. Ctrl (control); Glut (glutamate). Bands are cropped at the indicated size for better comprehension with ImageLab software (Bio-Rad, California, USA). **B** Primary cortical neurons from wild-type E18 pubs were exposed to the indicated concentration of CN03 3 h prior to 25 µM glutamate treatment for 24 h at DIV9. Data from *n* = 6 are shown as mean + SD. ^###^*P* < 0.001 compared to control; ***P < 0.001 compared to glutamate-treated control (ANOVA, Scheffé’s test). **C** Micromolar concentrations of glutamate stimulated excessive Ca^2+^ entry into neurons, a pathologic condition known as excitotoxicity. By application of CN03 protein, a known Rho activator, cofilin1 is deactivated via ROCK-LIMK pathways thereby promoting neuronal protection by circumventing cofilin1 activation and mitochondrial demise. NMDA-R *N*-methyl-d-aspartate receptor, ER endoplasmic reticulum, [Ca^2+^] intracellular calcium concentration, P Ser3-phosphorylation, ROCK Rho-associated serine/threonine kinase, LIMK LIM kinase, DNA deoxyribonucleic acid, roman numerals representing complex I–V of the respiratory chain.

As mentioned before, the phosphorylation status of cofilin1 Ser3 is considered to be a decisive determinant for actin binding. Since the Rho-ROCK pathway was identified to activate LIM domain kinase 1 and 2 (LIMK1, 2) [40], a crucial cofilin1-negative regulator, the Rho activator CN03 was administered for induction of cofilin1 Ser3 phosphorylation and thus deactivation of the protein. To validate the effect of 1 µg/mL CN03 exposure on cofilin1 phosphorylation, Western blot was performed using a specific antibody against phosphorylated Ser3-cofilin1. As clearly demonstrated, cofilin1 was dephosphorylated after glutamate exposure, whereas CN03 preserved the phosphorylation status (Fig. 6A). The effect of this manipulation was assessed in the MTT assay to quantify metabolically active cells. Interestingly, a 3-h pretreatment with 1 µg/mL CN03 rescued the loss of metabolic activity induced by 24-h exposure of glutamate (Fig. 6B). This beneficial effect was comparable to the potent NMDA-receptor antagonist MK801 (Fig. 6B).

### Administration of recombinant cofilin1 on isolated mitochondria impaired the mitochondrial membrane potential and respiration

**Fig. 7:**
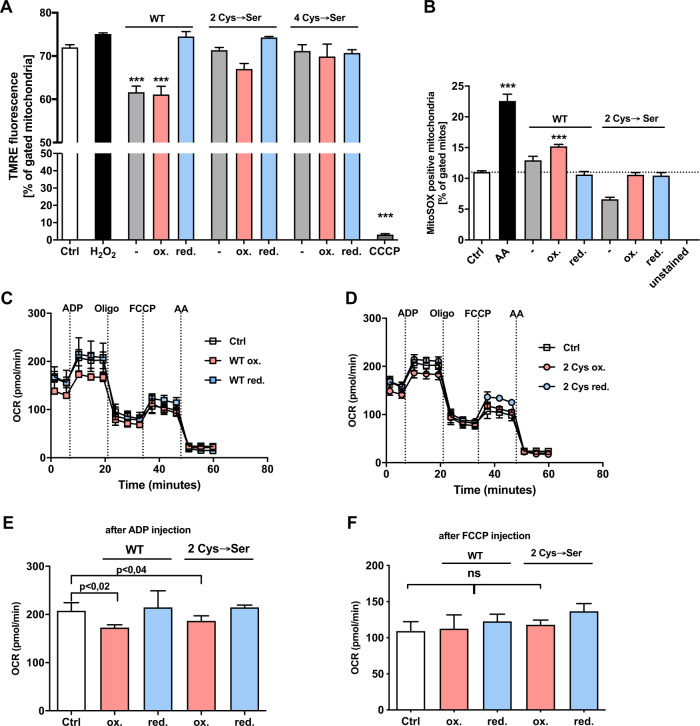
Direct effects of recombinant, oxidized cofilin1 impairs the mitochondrial membrane potential and respiration. **A** Recombinant cofilin1 protein was applied in the non-treated, the oxidized (100 µM H_2_O_2_) or in the reduced form (10 mM DTT). 75 µg mitochondria were incubated with 10 µg protein for 20 min at 37 °C and finally stained with TMRE (1:1000). 50 µM CCCP served as a positive control. 10,000 total events were measured and shown as mean + SD (*n* = 3 replicates). WT (wild-type cofilin1 protein); 2Cys (Cys139/Cys147 → serine mutation); 4Cys (39, 80, 139, 147 → serine mutation) ***P < 0.001 compared to ctrl (ANOVA, Scheffé’s test). **B** 75 µg mitochondria were incubated with 10 µg protein for 20 min at 37 °C and finally stained with MitoSOX red fluorescent dye (1:1000). 10 µM Antimycin A (AA) served as a positive control. In total, 10,000 total events were measured and shown as mean + SD (*n* = 3 replicates). WT (wild-type cofilin1 protein); 2Cys (Cys139/Cys147 → serine mutation); ***P < 0.001 compared to ctrl (ANOVA, Scheffé’s test). **C** In all, 10 µg mitochondria per well were incubated with the WT protein or **D** with the 2Cys mutant either in the native form, the oxidized form (100 µM H_2_O_2_) or in the reduced form (10 mM DTT) for 30 min at 37 °C and were administered to the Seahorse Analyzer. Mean + SD (*n* = 5–9 replicates). **E** Quantification of mitochondrial activity was conducted with the values delivered after the injection of ADP as a substrate for the OXPHOS phosphorylating capacity. **F** FCCP uncouples the oxygen consumption from ATP production and is used to assess maximal respiratory activity. ns not significant, WT wild-type cofilin1 protein, 2Cys Cys139/Cys147 → serine mutation. *P* values were calculated by ANOVA, Scheffé’s test.

Besides the well-established function of F-actin dynamics, cofilin1 has been linked to oxidative cell death, e.g., induced by the oxidant taurine chloramine [25] or H_2_O_2_ [41, 42]. Cofilin1 possesses several cysteine residues forming intra- or intermolecular disulfide bonds essential for the quaternary structure of the protein. In human cofilin1, four crucial cysteines have been described as prone to oxidation at positions 39, 80, 139, and 147 [27]. Dephosphorylation of Ser3 and oxidation of the aforementioned cysteine residues are considered as crucial prerequisites for mitochondrial localization after apoptosis induction [25]. We cloned different constructs to address the impact of specific cofilin1 mutants (2Cys: Cys139Ser/Cys147Ser; 4Cys: Cys39Ser, Cys80Ser, Cys139Ser, Cys147Ser). All recombinant proteins were stable at the temperature of 37 °C that was used for all experiments on isolated mitochondria (Supplementary Fig. 3S). The impact of recombinant cofilin1 was analyzed on mitochondrial superoxide formation, mitochondrial membrane potential, and mitochondrial respiration in mitochondria isolated from mouse brains. Strikingly, the reduced form of WT cofilin1 had no impact on the membrane potential, whereas the application of WT cofilin1 either in the non-treated form or in the oxidized state decreased the mitochondrial membrane potential. Further, the Cys139/147Ser mutation as well as the conversion of all four cysteines to serine completely abolished the effect of cofilin1 on isolated mitochondria (Fig. 7A). The ionophore CCCP was used to demonstrate the maximal effects of mitochondrial membrane collapse (Fig. 7A).

The maximal effect of mitochondrial superoxide generation was evoked by antimycin A treatment, a potent complex III-inhibitor of the respiratory chain (Fig. 7B). The oxidized form of the WT protein also induced a significant burst of mitochondrial superoxides, whereas the reduced WT protein and the 2Cys mutant generated comparable ROS levels to mitochondria of the untreated control group (Fig. 7B). The effect of the serine mutants on mitochondrial integrity and superoxide generation was therefore mainly attributed to the cysteine residues at positions 139 and 147.

Finally, to understand the effect of recombinant cofilin1 at a functional level, mitochondrial bioenergetics were evaluated using the Seahorse XFe96 Analyzer. The analysis of the ADP-driven mitochondrial activity revealed a significant impairment (*P* < 0.02) of the measured OCR of mitochondria challenged with the oxidized WT cofilin1 protein compared to the control condition (Fig. 7C–E). The oxidized form of the 2Cys mutant had a slightly minor derogating impact on the ADP-dependent respiration compared to the WT cofilin1 protein (WT *P* < 0.02 vs. 2Cys *P* < 0.04), which was completely reversed by reduction of these cysteine residues (Fig. 7D–F). The proton leak across the inner mitochondrial membrane or maximal respiration was not affected after the application of either the oxidized or reduced form of the recombinant proteins (Fig. 7C, D, F).

## Discussion

This study identified a pivotal role of cofilin1 upstream of mitochondrial damage in oxidative cell death induced by glutamate or erastin and in models of glutamate excitotoxicity in primary cortical neurons. Here, we demonstrate that cofilin1 downregulation in neuronal HT22 cells by specific cofilin1-targeting siRNA or by the genetic deletion in primary neurons exerts protective effects on mitochondrial function and cellular resilience. We have shown that cofilin1 deficiency affects mitochondrial superoxide accumulation, mitochondrial membrane potential, mitochondrial calcium accumulation, mitochondrial respiration, and ATP generation.

Mitochondrial superoxide production by complex I and III occurs during respiration. Moreover, it can either be a result of complex I defects, genetic abnormalities, or excessive mitochondrial calcium admission [43–45]. Massive mitochondrial calcium gathering induces the opening of the mitochondrial permeability transition pore and subsequent loss of the mitochondrial membrane potential leading to cell death (reviewed in ref. [46]). Our results emphasize that cofilin1 depletion can reduce massive mitochondrial calcium overload which may account for the protective mechanism upon cofilin1 depletion. In this regard, possible indirect effects by alteration of the actin cytoskeleton might be conceivable to impact mitochondrial calcium uptake, as previously discussed for INF2-knockout cells [47]. Mitochondrial ROS is especially detrimental for mitochondrial function, as it leads to mitochondrial DNA damage and subsequent impaired oxidative phosphorylation (OXPHOS) [48–50]. Our data show that the respiratory capacity of cofilin1-depleted cells exposed to erastin is reduced at earlier time points. The reduced respiratory capacity and reduced basal mitochondrial OCR may contribute positively to impaired mitochondrial ROS production and subsequent cell damage at the early stages of ferroptotic cell death. Therefore, preventing mitochondrial ROS accumulation by cofilin1 depletion is an efficient intervention point to retrieve neurons under pathophysiological conditions, as imposed here through glutamate- or erastin treatment.

The precise point of the detrimental action of cofilin1 was ascertained upstream of mitochondrial demise, but downstream of lipid peroxidation. Further, Annexin V/PI staining, xCELLigence measurement and MTT assay revealed that cell death was also attenuated by interfering with cofilin1 expression. However, neither significant activation of cofilin1 nor aberrant expression levels could be detected after exposure of the cells to glutamate or erastin, suggesting that oxidative stress and subsequent cell death is not executed via the alteration of cofilin1 phosphorylation or protein expression levels. Mitochondria are regarded as the point of no return upon cell death induction [51]; therefore, cofilin1 inhibition is efficient in preventing cell death in these paradigms of oxidative damage since cofilin1 is an important key player in death signaling upstream of mitochondria.

Excessive glutamate accumulation is a hallmark of several neurodegenerative diseases, stroke, and brain trauma [52, 53]. Cofilin1 was identified as a key player in different neurological diseases, e.g., in Alzheimer’s or Parkinson’s disease [54–57], and recent findings also implied a role for cofilin1 in ischemic brain damage [58, 59]. In this paradigm, cofilin1 phosphorylation was able to prevent detrimental cofilin-actin rod formation, thereby improving mitochondrial transport failure induced by oxygen and glucose deprivation [58]. In our study, CN03-induced phosphorylation also impressively protected neurons from glutamate-induced cell death revealing a potential target for future treatment strategies, conceivable for a variety of neurological disorders, such as stroke [58] or autism-like deficits [60]. A putative mechanism involves Rho activation by CN03, thereby phosphorylating cofilin1 via ROCK-LIMK pathways and finally promoting neuronal protection by circumventing cofilin1 activation and mitochondrial demise (Fig. 6C).

Underlining the strong effect of our loss-of-function approach, recent studies showed a direct effect of cofilin1 by binding to mitochondria [25, 61, 62]. It was demonstrated that cofilin1 gains activity to translocate to mitochondria upon oxidation by taurine chloramine, a physiological oxidant derived from neutrophils [25, 37]. In line with these results, our data suggest that cofilin1 is an important mediator upon oxidative distress, as cofilin1 depletion was sufficient to block mitochondrial damage and, thereby, the oxidative cell death cascades. Further, functional measurements in isolated mitochondria revealed a direct detrimental effect of cofilin1 on mitochondrial integrity and respiration. Cofilin1-mediated effects on mitochondria were first reported by Chua and coworkers in 2003 [26]. Later, four cysteine (39/80/139/147) and three methionine residues were identified, which are prone to oxidation, but only the oxidized cysteines were linked to mitochondrial demise [25]. In particular, a detrimental role for the oxidized form of cofilin1 upstream of mitochondria was unraveled in cell death models induced by the oxidants H_2_O_2_ or taurine chloramine (TnCl) [25, 63]. Detrimental mitochondrial transactivation of cofilin1 was even observed without any further stimulus when the cells express the oxidation-mimetic glycine residues at positions 39 or 80, respectively [63]. Apparently, cysteines do not only serve as redox sensors and mediate redox signaling, they are also crucial for the correct structural protein formation and interaction. Especially Cys39 and Cys80 can form intramolecular disulfide bonds and their oxidation mediated protein dephosphorylation (Ser3) after oxidation due to sterical effects [27]. Cys139 and Cys147 are able to form both, intra- and intermolecular disulfide bonds, thus presenting a prerequisite for cofilin1 oligomerization [64]. In the present study, specific mutations of either two (Cys139/147) or all four cysteine residues of the recombinant protein were realized to address the question of which specific cysteine residues contribute to the deleterious effects of the protein after oxidation. Intriguingly, the wild-type form of cofilin1 significantly decreased the mitochondrial membrane potential in isolated mitochondria. This detrimental effect was attenuated when cofilin1 residues at positions 139 and 147 were mutated to the non-oxidizable amino acid serine; and this effect was completely averted when all four cysteine residues were substituted by serine, suggesting cofilin1 oxidation can induce significant impairment of mitochondrial integrity and function. Accordingly, mitochondrial ROS accumulation was enhanced by the oxidized form of cofilin1 and, in line with the TMRE measurements, the 2Cys mutant did not exert mitochondrial ROS formation. These findings are in line with earlier findings on the Cys139/147 mutants of cofilin1 [25]. Further, the wild-type form of cofilin1 impaired oxygen consumption upon ADP injection, an indicator of complex II, III, and V-driven respiration. Although the mutation of Cys139 and 147 still led to a decrease of mitochondrial respiration, the effect was less pronounced compared to wild-type cofilin1.

In conclusion, the oxidation of Cys139 and Cys147 of cofilin1 are crucial in mediating the direct damage of mitochondria. However, the effects of the oxidized form of cofilin1 were always less pronounced compared to the positive controls (Antimycin A for mitochondrial-derived ROS and CCCP as an uncoupler to induce the collapse of the mitochondrial membrane potential). A possible explanation is delivered by Liu and coworkers who demonstrated that cofilin1 needs the interaction with p53 for strong impacts on mitochondrial function [62]. Overall, our data demonstrate a striking effect of cofilin1 in cell death mechanisms linked to oxidative distress, underlining that cofilin1 acts as a redox sensor in cell death mechanisms comprising oxytosis and ferroptosis. However, it is tempting to speculate that thiol switches of cofilin1 generally have also regulatory functions in physiological signal transduction pathways. Thus, our data suggest that interfering with cofilin1’s activity by pharmacological inhibition or imposing cofilin1 phosphorylation at serine residue 3 could provide new potential therapeutic strategies for neurodegenerative diseases in the future.

## Supplementary information

Supplement Figure Legends

Supplement Figure 1

Supplement Figure 2

Supplement Figure 3

## Data Availability

Supplementary information is available at the Cell Death and Disease website. The presented material is original research and is available in the original version on biorxiv.org: 10.1101/2020.09.09.289710.
