# Supplementary material for: Cofilin1 oxidation links oxidative distress to mitochondrial demise and neuronal cell death
Source: Cell Death Dis. 2021 Oct 16;12(11):953. doi: 10.1038/s41419-021-04242-1 (PMC8520533; doi:10.1038/s41419-021-04242-1)
Supplement: Supplementary file 1 — Supplement Figure Legends [file 41419_2021_4242_MOESM1_ESM.docx]

**Figure 1 S. Cofilin mRNA expression levels are not altered by glutamate or erastin challenge**

HT22 cells were treated with 5 mM glutamate or 0.5 µM erastin for the indicated time points following RNA isolation with subsequent cDNA synthesis. qPCR analysis was conducted using respective cDNA, normalizing cofilin to U6 (A) or GAPDH (B). Data are shown as mean + SD; n = 3 replicates. ns non-significant to Control (One Way-ANOVA, Bonferroni’s post-hoc test).

**Figure 2 S. Depletion of cofilin contributes to the resilience against erastin-mediated ferroptosis by reducing the respiratory capacity at early time points**

HT22 cells were transfected with two siRNAs against Cofilin1 at 15 nM for 24h. Scr siRNA was used as control. The cells were treated with erastin (0.5 µM) and the respective amount of DMSO for 6 hours following the analysis of the oxygen consumption rate (OCR) in a Seahorse XFe96 Analyzer. Data is shown as mean values ± SD (n = 8 replicates).

**Figure 3 S. Expression, purification and thermal stability of recombinant cofilin1**

Recombinant WT, 2Cys→ Ser and 4Cys→ Ser Cofilin-1 were expressed in E. coli and purified using the IMAC principle. **A** Expression samples prior (-) and after induction (+) with Isopropyl β-D-1-thiogalactopyranoside (IPTG). **B** Purified proteins were analyzed by SDS-Page and Coomassie staining. The exemplary illustration shows WT Cofilin1 elution fractions 1-4 with a molecular weight of 18 kDa. **C** Proteins were incubated at increasing temperatures and thermal stability was determined photometrically at 600 nm.
