# Supplementary figures and images for: Cofilin1 oxidation links oxidative distress to mitochondrial demise and neuronal cell death
Source: Cell Death Dis. 2021 Oct 16;12(11):953. doi: 10.1038/s41419-021-04242-1 (PMC8520533; doi:10.1038/s41419-021-04242-1)

**Fig. 1 S**

**A**

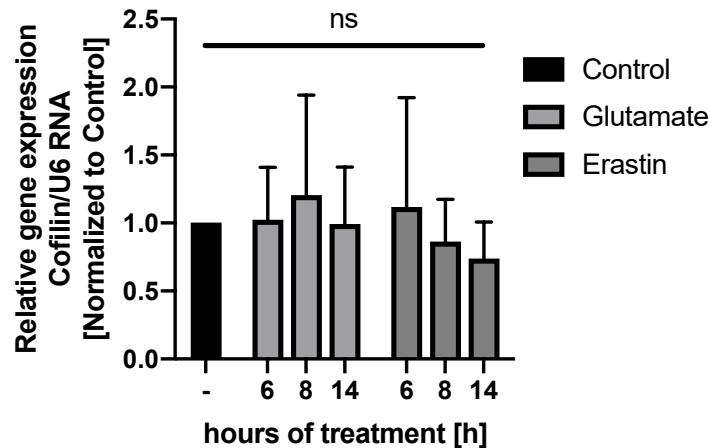

**B**

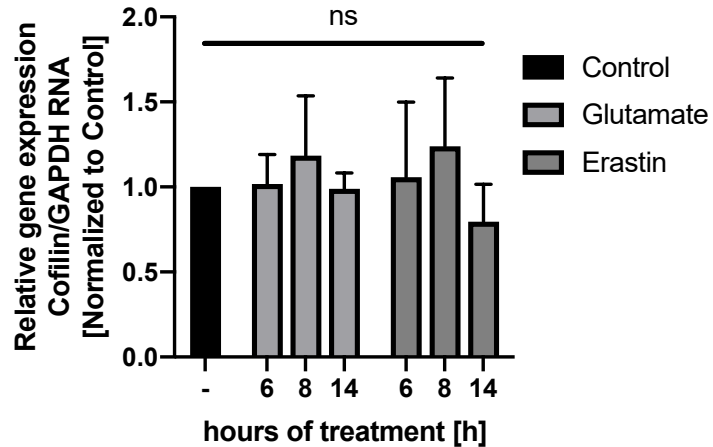

Supplement: Supplementary file 2 — Supplement Figure 1 [file 41419_2021_4242_MOESM2_ESM.pdf]

**Fig. 2 S**

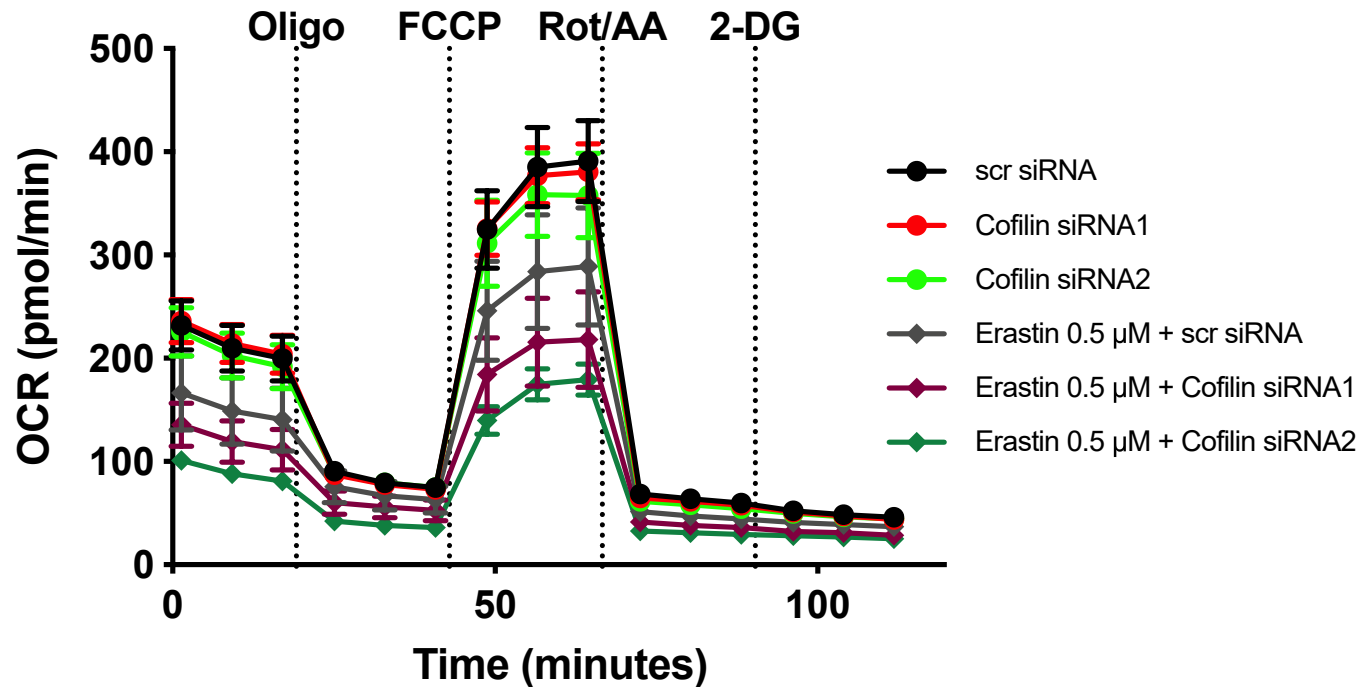

Supplement: Supplementary file 3 — Supplement Figure 2 [file 41419_2021_4242_MOESM3_ESM.pdf]

**Fig. 3 S**

**A**    -    +

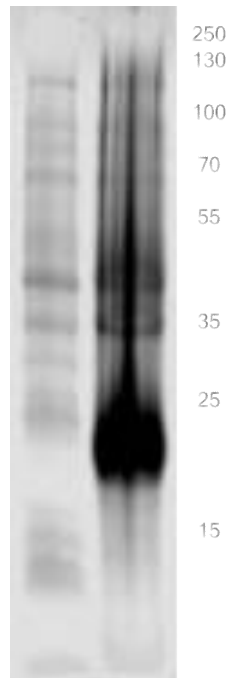

**B**

1    2    3    4

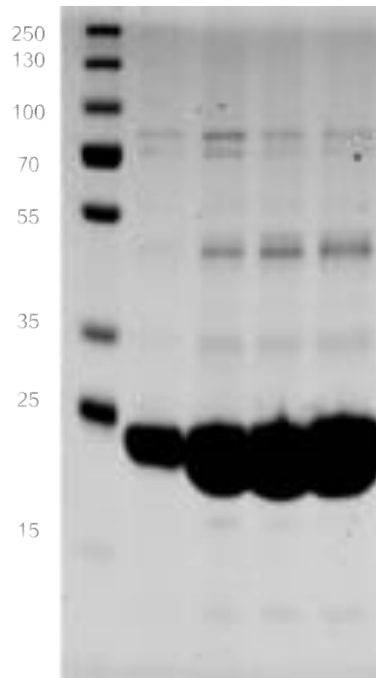

**C**

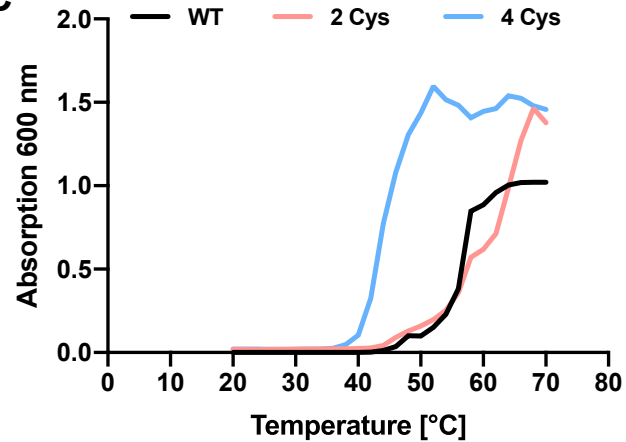

Supplement: Supplementary file 4 — Supplement Figure 3 [file 41419_2021_4242_MOESM4_ESM.pdf]
